# Effect of reduced nutritional supply on the metabolic activity and survival of cariogenic bacteria *in vitro*

**DOI:** 10.1080/20002297.2019.1605788

**Published:** 2019-04-22

**Authors:** Petra Ganas, Falk Schwendicke

**Affiliations:** Department of Operative and Preventive Dentistry, Charité – Universitätsmedizin Berlin, Berlin, Germany

**Keywords:** Dental caries, selective excavation, Streptococcus sobrinus, Actinomyces naeslundii, Lactobacillus rhamnosus, carbon metabolism, stress adaptation, survival rate

## Abstract

Sealed cariogenic bacteria are deprived from dietary carbohydrate, but could be provided with nutrients by pulpal fluids, with adaptive strain-specific activities being possible. We investigated survival and metabolic activity of the cariogenic bacteria *Streptococcus sobrinus, Actinomyces naeslundii* and *Lactobacillus rhamnosus* in different carbohydrate-limited media without carbon source (CLM), or containing glucose (CLM-G), albumin (CLM-A), or α1-acid glycoprotein (CLM-AGP) *in vitro*. Bacterial metabolite concentrations (lactate, pyruvate, oxaloacetate, citrate, acetate, formate, ethanol, acetoin) after 20 and 4 hours incubation, and bacterial numbers (CFU) after 24 hours incubation were analyzed using multivariate-analysis-of-variance (MANOVA). The medium (p = 0.02/MANOVA), strain and incubation-time (both p < 0.001) had significant impact on metabolite concentrations. Bacteria secreted mainly lactate (80.3 µg/10^6^ bacteria *S. sobrinus*) and acetate (54.5 µg/10^6^ bacteria *A. naeslundii*). Nearly all metabolites were produced in higher concentrations in *S. sobrinus* than in *A. naeslundii* or *L. rhamnosus* (p < 0.05/HSD). Metabolite concentration was significantly higher in CLM-G than in other media for most metabolites (p < 0.05). *L. rhamnosus* showed significantly lower survival than *S. sobrinus* and *A. naeslundii* (p < 0.05/HSD) regardless of the media, while *S. sobrinus* and *A. naeslundii* showed medium-specific survival. Survival of carbon starvation was strain- and medium-specific. Sustained organic acid production was found for all strains and media.

## Introduction

Bacteria are the prime etiological agent in development and progression of dental caries, which is why caries management has long focused on controlling cariogenic bacteria. One such therapy for controlling the activity of cariogenic bacteria is sealing them, with sealed bacteria being cut off from the supply with dietary carbohydrates, impacting on bacterial survival, metabolic activity, and resulting in clinical lesion arrest [1,2]. Even for deep carious lesions, sealing carious dentin and, with it, cariogenic bacteria in proximity to the dental pulp is recommended in order to avoid exposure and irritation of the pulp [3–5].

By sealing, bacteria are hence shifted into a new ecological niche with extensive starvation stress. However, in deeper lesions close to the dental pulp, bacteria could be provided with nutrients by the pulpal fluid over the dentinal tubules. This fluid consists of different compounds like glucose, glycoproteins and proteins [6,7], which can function as carbon sources.

Because the survival rate of sealed bacteria has an influence on pulp survival and hence therapy success, the aim of the present study was to investigate the bacterial potential to adapt to limited nutritional conditions in order to survive and maintain metabolic activity. An *in vitro* cultivation system with well-defined carbohydrate-limited media (CLM) with different carbohydrate sources was established to analyze the metabolic and growth behavior of three different cariogenic bacterial strains. Then, metabolites, mainly organic acids, produced and secreted by the bacteria were examined alongside bacterial survival. Our hypothesis was that survival and metabolic activity differed between species, and was significantly influenced by the provided carbohydrates sources.

## Materials and methods

### Bacteria and growth conditions

The bacterial strains *Streptococcus sobrinus*, DSM 20742, *Actinomyces* naeslundii, DSM 43013 and *Lactobacillus rhamnosus*, DSM 20021 (DMSZ, Braunschweig, Germany) were used in the present study. The strains *S. sobrinus* and *A. naeslundii* were maintained on blood agar plates COLS+ (Oxoid, Wesel, Germany) whereas the *L. rhamnosus* strain was cultured on deMan-Rogosa-Sharpe (MRS) agar (Oxoid) at 37°C for 2–3 days under aerobic conditions.

The bacteria were precultured in brain-heart-infusion (BHI) broth (Carl Roth, Karlsruhe, Germany) for 18 h at 37°C aerobically in tightly closed 15 ml Falcon tubes (Corning, Kaiserslautern, Germany). Afterwards the bacteria were transferred into BHI containing 1% glucose (Carl Roth) and 1% sucrose (Carl Roth) with an inoculum (mean ± SD) of 6 ± 3*10^5^ cells as 1 ml-cultures in 1.5 ml Eppendorf tubes (Eppendorf, Hamburg, Germany), and incubated at 37°C aerobically for further 18 h. After centrifugation of the cultures at 10,600 g for 5 min at room temperature, the supernatants were removed and 1 ml CLM (see below) was pipetted into each tube.

The composition of CLM was based on the semi-defined medium described by Homer et al. [8] and consisted of 20.2 mM anhydrous KH_2_PO_4_ (all Carl Roth unless otherwise outlined), 23.9 mM Na_2_HPO_4_*2H_2_O (Merck, Darmstadt, Germany), 0.2 mM NaCl, 72.2 mM NaCH_3_COO*3H_2_O, 4.5 mM (NH_4_)_2_SO_4_, 0.9 mM Na_3_C_6_H_5_O7*2H_2_O (Merck), 0.2 mM adenine, 0.3 mM uracil, 0.1 mM guanine HCl, 0.07 mM FeSO_4_*7H_2_O, 0.07 mM MnSO_4_*H_2_O, 2.1 mM MgSO_4_*7H_2_O, 14.0 mM L-cysteine HCl (Sigma Aldrich, Taufkirchen, Germany) and 3.8 mM Na_2_CO_3_ (Merck). The CLM was adjusted to pH 7.0 and supplemented with substances, which served as energy sources for the bacteria, creating four different CLM variations: CLM without energy source, CLM with 0.01% glucose (Carl Roth) (CLM-G), CLM with 0.01% albumin fraction V (Carl Roth) (CLM-A) and CLM with 0.01% α1-acid glycoprotein from bovine plasma (Sigma Aldrich) (CLM-AGP).

The bacterial cultures were incubated at 37°C aerobically for 20 h, constituting a phase of bacterial adaptation to the altered conditions, followed by a centrifugation step at 10,600 g for 5 min at room temperature in order to collect the supernatants. The supernatants were centrifuged a second time at 20,800 g for 5 min at room temperature and then deproteinized using Amicon Ultra centrifugal filters 0.5 ml with a 10 kDa molecular weight cut-off (MWCO) (Merck) at 10,000 g for 10 min at room temperature. The deproteinized supernatants were stored at −80°C until later processing.

The bacterial cells were cultured for further 4 h at 37°C aerobically after the addition of 1 ml fresh CLM into each Eppendorf tube as before. Thereafter, the cultures were centrifuged and the supernatants were collected, deproteinized and stored as described above. Medium exchange after 20 h of incubation was performed in order to simulate flux of nutrients and to ensure constant metabolism of bacterial cells.

The pH of all deproteinized supernatants was measured with MColorpHast pH-indicator strips pH 2.0–9.0, pH 4.0–7.0 and pH 6.5–10.0 (Merck).

The growth experiments were performed in triplicates for each CLM variation. Control samples without bacterial cells were set up using the medium BHI with 1% glucose and 1% sucrose followed by the four different CLM variations processed in the same way as the bacterial cultures.

### Metabolite assays

The metabolite contents of lactate, pyruvate, oxaloacetate, citrate, acetate, formate and ethanol in the deproteinized culture supernatants were measured by colorimetric assay kits (Sigma Aldrich) according to the protocols of the manufacturer. The content of acetoin in the supernatants was detected based on the assay method described by Romick and Fleming [9]. The acetoin standard curve series contained 0, 0.25, 0.5, 0.75, 1.0, 1.25, 1.5, 1.75, 2.0, 2.25, 2.5, 2.75 and 3.0 mM acetoin (Sigma Aldrich). In order to detect the acetoin content by a colorimetric reaction, 5 µl of O’Meara’s Reagent (Sigma Aldrich), 5 µl of 1% L-arginine solution (Carl Roth) and 3 µl Barritt’s Reagent A (Sigma Aldrich) were added to 50 µl of each deproteinized supernatant sample and to 50 µl of acetoin standard curve series. After incubation at 37°C for 30 min, the absorbance was measured at 490 nm. Measurements of the absorbance were performed with the 96 well plate spectrophotometer Multiskan Go (Thermo Scientific, Schwerte, Germany). As the CLM contained 72.2 mM acetate and 0.9 mM citrate, and no other metabolites, changes in metabolite concentration were determinable.

### Bacterial survival

At the end of the incubation for 20 h plus 4 h (i.e. after 24 h) in CLM, the culture supernatants were removed and the bacterial cells resuspended in 1 ml of 0.9% NaCl solution per tube by scraping and dispersing the cell material with the help of inoculating needles (Simport, Beloeil, Canada) and a vortex mixer. Serial dilutions of the bacterial suspensions were prepared and 100 µl aliquots of them were split on COLS+ agar plates for *S. sobrinus* and *A. naeslundii* or on MRS agar plates for *L. rhamnosus*. After incubation at 37°C for 2–3 days under aerobic conditions, the colonies were counted and the colony forming units (CFU) per sample were calculated.

In order to determine the initial cell count (t = 0) bacterial strains were precultured in BHI for 18 h at 37°C aerobically in 15 ml Falcon tubes. Then bacteria were transferred into BHI containing 1% glucose and 1% sucrose with an inoculum of 6 ± 3*10^5^ cells as 1 ml-cultures in 1.5 ml Eppendorf tubes, incubated at 37°C aerobically for further 18 h. Afterwards the samples were centrifuged, the culture supernatants were removed and the bacterial cells were resuspended into 1 ml of 0.9% NaCl solution. Plate counting was performed as described above and CFUs per sample were calculated.

### Statistical analysis

Descriptive analysis was performed using means and standard deviations, stratified according to strain and medium. Multivariate analysis of variance (MANOVA) with post-hoc Tukey’s honestly significant difference test (HSD) was performed for statistical analysis. Differences with p < 0.05 were considered to be statistically significant.

## Results

The metabolic analyses of the strains *S. sobrinus, A. naeslundii* and *L. rhamnosus* grown in CLM, CLM-G, CLM-A and CLM-AGP displayed a distinct profile of extracellular concentration changes of different metabolic products for each bacterial strain (Table 1). The medium (p = 0.02/MANOVA), the strain (p < 0.001) and the incubation time (p < 0.001) all had significant impact on the metabolite concentrations. All bacteria produced and secreted mainly lactate and acetate. Nearly all metabolites were produced in higher concentrations in *S. sobrinus* than in *A. naeslundii* or *L. rhamnosus* (p < 0.05/HSD). The metabolite concentration was significantly higher in CLM-G than in other media for most metabolites (p < 0.05). Details for the statistical analysis of metabolite concentrations can be found in Table 2.

**Table 1.** Changes in metabolite concentration compared with baseline in the culture supernatants after incubation for 20 h and another 4 h. Negative values indicate uptake of metabolites by bacteria. Bacterial strains were cultivated in carbohydrate-limited medium without carbon source (CLM) and in medium with glucose (CLM-G), albumin (CLM-A) or α1-acid glycoprotein (CLM-AGP). Data from three independent experiments are given as means ± standard deviations. The statistical evaluation is provided in Table 2.

			Metabolites (µg/10^6^ bacterial cells)							
Bacterial strain	Minimal medium	Incubat. time	Lactate	Pyruvate	Oxaloacetate	Citrate	Acetate	Formate	Ethanol	Acetoin
*S. sobrinus*	CLM	20 h	23.04 ± 7.02	1.03 ± 0.59	0.59 ± 0.14	1.37 ± 0.41	7.36 ± 11.32	0.12 ± 0.03	0.53 ± 0.19	0.15 ± 0.09
		4 h	0.03 ± 0.01	nd	0.01 ± 0.04	0.17 ± 0.04	−21.83 ± 33.72	nd	nd	nd
	CLM-G	20 h	80.25 ± 9.50	2.53 ± 0.31	2.66 ± 0.69	4.61 ± 0.66	−139.39 ± 40.61	0.47 ± 0.06	1.71 ± 0.27	1.86 ± 0.26
		4 h	0.45 ± 0.06	nd	0.17 ± 0.06	3.31 ± 0.58	−45.19 ± 68.19	0.06 ± 0.02	nd	nd
	CLM-A	20 h	16.57 ± 5.61	0.67 ± 0.22	0.36 ± 0.10	1.10 ± 0.41	9.94 ± 13.65	0.11 ± 0.04	0.38 ± 0.11	0.54 ± 0.26
		4 h	0.02 ± 0.01	nd	0.04 ± 0.03	0.41 ± 0.25	−8.89 ± 2.58	nd	0.03 ± 0.07	nd
	CLM-AGP	20 h	15.68 ± 1.98	0.60 ± 0.11	0.34 ± 0.03	0.86 ± 0.14	4.03 ± 5.49	0.09 ± 0.01	0.34 ± 0.04	0.61 ± 0.11
		4 h	0.01 ± 0.01	nd	0.07 ± 0.01	0.30 ± 0.15	−0.68 ± 10.11	nd	nd	nd
*A. naeslundii*	CLM	20 h	13.95 ± 5.56	1.04 ± 0.38	nd	0.27 ± 0.08	−8.47 ± 23.04	1.67 ± 0.68	0.41 ± 0.15	nd
		4 h	0.03 ± 0.01	nd	0.07 ± 0.05	0.63 ± 0.34	−34.54 ± 11.24	nd	0.17 ± 0.13	0.32 ± 0.53
	CLM-G	20 h	20.34 ± 12.40	1.58 ± 1.03	nd	−0.24 ± 0.19	54.53 ± 45.67	2.53 ± 1.69	0.52 ± 0.35	0.29 ± 0.62
		4 h	0.02 ± 0.01	nd	0.32 ± 0.23	−1.35 ± 0.90	−32.22 ± 40.67	nd	0.06 ± 0.08	1.32 ± 0.81
	CLM-A	20 h	17.19 ± 8.98	1.05 ± 0.46	nd	−1.25 ± 0.77	−130.51 ± 64.36	1.89 ± 0.89	0.46 ± 0.23	2.01 ± 0.96
		4 h	0.11 ± 0.06	nd	0.24 ± 0.12	−0.72 ± 0.88	−74.43 ± 49.52	nd	0.19 ± 0.10	nd
	CLM-AGP	20 h	5.94 ± 2.09	0.38 ± 0.16	nd	−0.16 ± 0.07	20.10 ± 1.74	0.56 ± 0.22	0.17 ± 0.05	nd
		4 h	0.01 ± 0.01	nd	0.04 ± 0.03	−0.01 ± 0.07	−11.11 ± 10.00	nd	0.02 ± 0.18	nd
*L. rhamnosus*	CLM	20 h	0.53 ± 0.04	nd	nd	0.08 ± 0.02	−1.00 ± 1.23	0.001 ± 0.001	0.01 ± 0.002	nd
		4 h	0.001 ± 0.001	nd	0.001 ± 0.005	0.04 ± 0.01	3.17 ± 0.62	nd	nd	nd
	CLM-G	20 h	0.95 ± 0.46	0.003 ± 0.001	nd	0.06 ± 0.03	1.09 ± 1.01	0.001 ± 0.001	0.01 ± 0.001	nd
		4 h	0.02 ± 0.01	nd	0.004 ± 0.001	−0.10 ± 0.03	−1.63 ± 5.95	nd	nd	nd
	CLM-A	20 h	0.36 ± 0.04	0.004 ± 0.001	0.001 ± 0.004	−0.01 ± 0.01	−5.24 ± 0.77	0.001 ± 0.001	0.01 ± 0.001	0.09 ± 0.008
		4 h	0.001 ± 0.001	nd	0.01 ± 0.004	0.02 ± 0.03	−1.77 ± 0.37	nd	nd	nd
	CLM-AGP	20 h	0.54 ± 0.10	0.004 ± 0.005	0.01 ± 0.006	−0.01 ± 0.01	3.16 ± 1.14	0.001 ± 0.001	0.02 ± 0.01	nd
		4 h	0.02 ± 0.001	nd	0.003 ± 0.001	0.01 ± 0.01	−1.52 ± 2.05	nd	nd	nd

nd, not detectable.

**Table 2.** Statistical analysis of data for metabolite concentrations in the culture supernatants after incubation for 20 h and another 4 h. The bacterial strains *Streptococcus sobrinus* (SS), *Actinomyces naeslundii* (AN) and *Lactobacillus rhamnosus* (LR) were cultivated in carbohydrate-limited medium without carbon source (CLM) and in medium with glucose (CLM-G), albumin (CLM-A) or α1-acid glycoprotein (CLM-AGP).

	Bacterial strain		Carbohydrate-limited medium		Incubation time
Metabolite	MANOVA p value	Tukey’s HSD homogeneous groups	MANOVA p value	Tukey’s HSD homogeneous groups	MANOVA p value
Lactate	**0.001**	LR_a_ AN_a_ SS_b_	**0.019**	CLM_a_ CLM-G_b_ CLM-A_a_ CLM-AGP_a_	**0.001**
Pyruvate	**0.001**	LR_a_ AN_b_ SS_b_	**0.026**	CLM_ab_ CLM-G_b_ CLM-A_ab_ CLM-AGP_a_	**0.001**
Oxaloacetate	**0.001**	LR_a_ AN_a_ SS_b_	**0.018**	CLM_a_ CLM-G_b_ CLM-A_ab_ CLM-AGP_a_	**0.031**
Citrate	**0.001**	LR_a_ AN_a_ SS_b_	**0.006**	CLM_ab_ CLM-G_b_ CLM-A_a_ CLM-AGP_a_	0.152
Acetate	0.134	LR_a_ AN_a_ SS_a_	0.110	CLM_a_ CLM-G_a_ CLM-A_a_ CLM-AGP_a_	0.744
Formate	**0.001**	LR_a_ AN_b_ SS_a_	0.333	CLM_a_ CLM-G_a_ CLM-A_a_ CLM-AGP_a_	**0.001**
Ethanol	**0.001**	LR_a_ AN_b_ SS_b_	**0.036**	CLM_ab_ CLM-G_b_ CLM-A_ab_ CLM-AGP_a_	**0.001**
Acetoin	**0.013**	LR_a_ AN_b_ SS_b_	**0.025**	CLM_a_ CLM-G_a_ CLM-A_a_ CLM-AGP_a_	**0.021**

Data were assessed for statistically significant effects of independent variables (bacterial strains, culture media, incubation times) on dependent variable (metabolite contents) using MANOVA and post-hoc Tukey’s HSD test. Significant differences overall groups are indicated in bold and homogeneous subsets between groups by shared subscript letters. Differences with p < 0.05 were considered to be statistically significant (HSD-test).

In detail, *S. sobrinus* preferentially formed lactate, whereas *A. naeslundii* predominantly produced acetate, in both cases with the highest contents for the cultivation in CLM-G (*S. sobrinus* with 80.3 µg lactate/10^6^ bacterial cells and *A. naeslundii* with 54.5 µg acetate/10^6^ bacterial cells). For *L. rhamnosus* the measured values of lactate (values <1 µg/10^6^ bacterial cells) and acetate (values <4 µg/10^6^ bacterial cells) were low in all four variations of the CLM. The metabolites pyruvate, oxaloacetate, citrate, formate, ethanol and acetoin reached only low concentrations in all media. There was an uptake of extracellular acetate from the culture medium by all three bacterial strains, which was highest for *S. sobrinus* cultivated in CLM-G (concentration decreased by 139.4 µg/10^6^ bacterial cells) and *A. naeslundii* grown in CLM-A (concentration decreased by 130.5 µg/10^6^ bacterial cells). *A. naeslundii* also ingested citrate in CLM-G, CLM-A and CLM-AGP. The pH values of the cultures remained unchanged compared to the start pH value (pH 7.0) for all tested bacterial strains in the four CLM variations during the whole incubation period (20 h plus 4 h).

**Figure 1.**
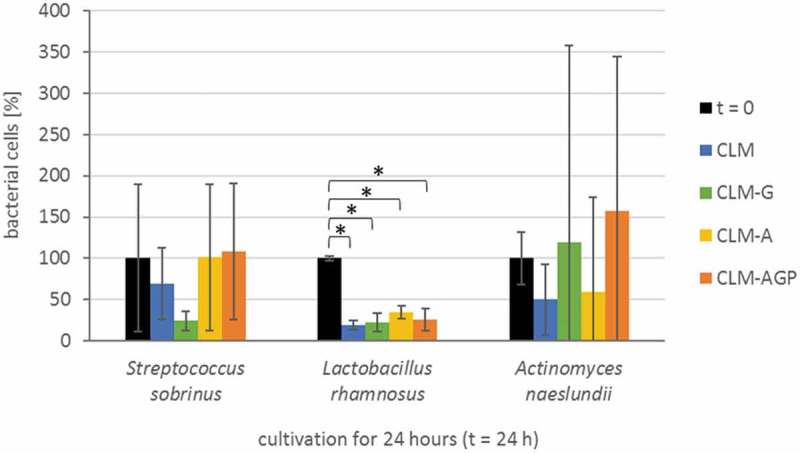
Bacterial survival. The relative number of colony-forming units (CFU) per sample in different culture media (color-coded); carbohydrate-limited medium without carbon source (CLM) and in carbohydrate-limited medium with glucose (CLM-G), albumin (CLM-A) or α1-acid glycoprotein (CLM-AGP) at the start (t = 0) and at the end of incubation after 20 + 4 h (t = 24 h). The initial cell counts at t = 0 were regarded as 100%. The means obtained from three independent experiments are given. Error bars indicate standard deviations. *Statistical significance between pairs showing differences with p < 0.05 (HSD-test).

The results of the bacterial survival analysis is shown in Figure 1. Survival differed significantly between strains; *L. rhamnosus* showed significantly lower survival than *S. sobrinus* and *A. naeslundii* (p < 0.05/HSD). Survival was not significantly different between media (p > 0.05). In detail, under the used growth conditions the bacterial strains *S. sobrinus* and *A. naeslundii* were grown as homogenous biofilms, whereas *L. rhamnosus* mainly formed cell pellets. After 24 h, bacterial cell numbers were significantly reduced for *L. rhamnosus* by up to 81% in all four test media. In all remaining media, no significant changes in cell counts were detected.

## Discussion

The present *in vitro* study tested the metabolic activity and survival of the cariogenic bacteria *S. sobrinus, A. naeslundii* and *L. rhamnosus* under different environmental stress conditions. We found bacteria to exhibit a relevant, strain-specific potential to stay viable under reduced and restricted nutritional conditions, and also to exert adapted metabolic activity in parallel. Our study confirms findings from *in vivo* studies, which demonstrated long-term survival of sealed bacteria [10,11]. Strain-specific survival was also demonstrated *in vitro* recently [12].

The collected data indicate that the bacterial strains made use of different strategies in order to adapt to the environmental changes. Growth under high-carbon conditions allows lactic acid bacteria to produce and accumulate intracellular polysaccharides [13–16]. Bacterial cells can use these glycogen-like storage polymers to overcome starvation periods. In this regard the formation and secretion of lactate in great amounts by the strains *S. sobrinus* and *A. naeslundii* during the cultivation for the first incubation period in all four low-carbon variations of CLM can be explained by the consumption of such intracellular storage polysaccharides.

Under the tested cultivation conditions, all three bacterial strains performed a heterofermentative metabolism. In addition to lactate, acetate was formed as a second main end product. Bacteria can use different pathways to degrade the intermediate pyruvate to gain energy (ATP), to regulate the intracellular reduction-oxidation balance (NADH/NAD ratio) and thereby to optimize the metabolic output. Therefore, lactate dehydrogenase, the predominantly active enzyme under sugar excess conditions, was substituted during nutritional depletion by pyruvate formate lyase (PFL, anaerobic condition) and pyruvate dehydrogenase (PDH, aerobic condition). Afterwards, the produced acetyl-CoA was further transformed into acetate or ethanol, both substances determined in the culture media [15,17–20]. Moreover, *S. sobrinus* also used acetyl-CoA to produce citrate. This finding is in agreement with the investigations of oral bacteria by Takahashi et al. [21] and Norimatsu et al. [22]. The ability of the bacterial strains to use the PFL pathway was confirmed by the detection of secreted formate.

Aside from fermentation of carbon sources with regard to cell proliferation, lactic acid bacteria also produce flavor compounds [20,23,24]. This could be demonstrated for *S. sobrinus* and *A. naeslundii* under the tested conditions by observing low levels of acetoin in some of their cultures.

The prolonged cultivation of the bacterial strains in nutrient-poor CLM resulted in a metabolic switch to a scavenging mode seeking for alternative carbon sources. Therefore, it seems to be coherent that all three bacterial strains ingested acetate from the culture media. Pathways for acetate assimilation via the acetate:CoA ligase or the reverse reaction of the phosphotransacetylase-acetate kinase are described for *Escherichia coli* [25]. Similar reactions are conceivable also for lactic acid bacteria.

In addition to acetate, the strains *A. naeslundii* and *L. rhamnosus* showed an uptake of citrate. The transport of citrate is driven by citrate:metal symporters described as co-transport with Ca^2+^ in *Lactobacillus casei* [26] and Fe^3+^ in *Streptococcus mutans* [27]. Intracellularly, the enzymes citrate lyase and oxaloacetate decarboxylase convert citrate into pyruvate and CO_2_ [26–28].

Furthermore, one should consider that lactic acid is not only a metabolic end product. Lactic acid bacteria can also utilize lactate as a carbon source by conversion into pyruvate using two different enzymes, a lactate oxidase or a NAD-independent lactate dehydrogenase [15,29,30]. Jyoti et al. [31] described for the cultivation of *L. rhamnosus* in a glucose-containing medium a diauxic growth profile, with a second growth phase on the fermentation product lactic acid. This metabolic feature might explain why only very low lactate contents could be detected for the *L. rhamnosus* grown in CLM.

Lactobacilli also strictly regulate their gene expression in response to sugar starvation [32,33]. Carbohydrate depletion leads to an extensive suppression of cellular pathways. The low amounts of metabolites detected in all *L. rhamnosus* cultures indicate that this bacterial strain exhibited a similar stress behavior. The bacteria seem to enter a physiological state of reduced metabolic activity. Parry et al. [34] demonstrated that such a dormant state can be achieved by changes of the bacterial cytoplasm. In this way, bacteria improve their stress-resistance and long-term survival under adverse environmental conditions.

The collected data regarding the cell numbers in the cultures clearly show the potential of the bacteria to adapt and to survive periods of carbon starvation. Viable cells were determined for all three bacterial strains grown in the four tested variations of CLM. For some cultivation conditions, no significant changes of the cell numbers were found at all, while in others, there was a reduction of bacterial cells. Notably, in a clinical setting, carious lesions will contain a far larger spectrum of bacterial strains [35], which could have a supportive effect on the survival rate of these bacteria, for example by cross-feeding etc.

Overall, the sustained survival and organic acid production by sealed bacteria may have clinical relevance, as acids can further demineralize the dentin, but also affect pulpal health. As clinical studies are only limitedly able to capture detailed pulp histological changes, *in vitro* simulations may be advisable in this direction. Notably, we did not detect pH decreases in the media; this, however, may be ascribed to experimental conditions (the media were buffering the acids). Further tests, involving long-term cultivation of bacteria (to test their demineralization capability, but also long-term adaption to stress) are advisable. Understanding bacterial adaptation processes to acquire stress-resistance and identifying the underlying molecular mechanisms may help to develop targeted supplementary treatment approaches for sealing carious lesions.

This study has a number of limitations. As discussed, investigation of mono-species cultures are suited to gain insights into strain-specific metabolic behavior of bacteria, while in nature microbiocoenoses are common. The clinical transferability of our findings may hence be limited. To gain a more profound and clinically applicable understanding, co-cultivation studies with several strains are advisable. A second limitation is predicated by our selection of performed metabolic assays, which was focused on carbon sources. Because the dental pulpal fluid contains also proteins, it would be interesting to investigate how intensively bacteria utilize these compounds and which metabolic products are formed and secreted. Last, the relative short cultivation time used in the study limits the impact of the present survival data. Longer cultivation period may allow to capture more nuanced differences between the survival potentials of cariogenic bacterial strains, in addition to the possible benefits discussed above.

In conclusion and within these limitations, all investigated cariogenic bacteria exhibited a relevant potential to survive nutrient (carbon source) starvation. In order to achieve this, bacteria adapted their metabolism, using strain-specific strategies like degradation of intracellular storage polymers, switches between metabolic pathways, usage of alternative carbon sources and reduction of their metabolic activity. During sugar starvation, lactic acid bacteria benefit from their metabolic flexibility.
